# Contra-Directional Expression of Plasma Superoxide Dismutase with Lipoprotein Cholesterol and High-Sensitivity C-reactive Protein as Important Markers of Parkinson’s Disease Severity

**DOI:** 10.3389/fnagi.2020.00053

**Published:** 2020-03-06

**Authors:** Wanlin Yang, Zihan Chang, Rongfang Que, Guomei Weng, Bin Deng, Ting Wang, Zifeng Huang, Fen Xie, Xiaobo Wei, Qin Yang, Mengyan Li, Kefu Ma, Fengli Zhou, Beisha Tang, Vincent C. T. Mok, Shuzhen Zhu, Qing Wang

**Affiliations:** ^1^Department of Neurology, Zhujiang Hospital of Southern Medical University, Guangzhou, China; ^2^Department of Neurology, The First People Hospital of Zhaoqing, Zhaoqing, China; ^3^Department of Neurology, Guangzhou First People’s Hospital, Guangzhou, China; ^4^Department of Neurology, Shenzhen People Hospital, Shenzhen, China; ^5^Department of Respiratory Medicine, The Third Affiliated Hospital of Sun Yat-Sen University, Guangzhou, China; ^6^Department of Neurology, Xiangya Hospital of Central South University, Changsha, China; ^7^Gerald Choa Neuroscience Centre, Department of Medicine and Therapeutics, Faculty of Medicine, Prince of Wales Hospital, The Chinese University of Hong Kong, Shatin, China

**Keywords:** superoxide dismutase, high-sensitivity C-reactive protein, lipoprotein cholesterol, Parkinson’s disease, inflammation, oxidative stress

## Abstract

**Aim**: Oxidative stress and inflammation play critical roles in the neuropathogenesis of PD. We aimed to evaluate oxidative stress and inflammation status by measuring serum superoxide dismutase (SOD) with lipoprotein cholesterol and high-sensitivity C-reactive protein (hsCRP) respectively in PD patients, and explore their correlation with the disease severity.

**Methods**: We performed a cross-sectional study that included 204 PD patients and 204 age-matched healthy controls (HCs). Plasma levels of SOD, hsCRP, total cholesterol, high-density lipoprotein cholesterol (HDL-C) and low-density lipoprotein cholesterol (LDL-C) were measured. A series of neuropsychological assessments were performed to rate the severity of PD.

**Results**: The plasma levels of SOD (135.7 ± 20.14 vs. 147.2 ± 24.34, *P* < 0.0001), total cholesterol, HDL-C and LDL-C in PD were significantly lower than those in HCs; the hsCRP level was remarkably increased in PD compared to HC (2.766 ± 3.242 vs. 1.637 ± 1.597, *P* < 0.0001). The plasma SOD was negatively correlated with the hsCRP, while positively correlated with total cholesterol, HDL-C, and LDL-C in PD patients. The plasma SOD were negatively correlated with H&Y, total UPDRS, UPDRS (I), UPDRS (II), and UPDRS (III) scores, but positively correlated with MoCA and MMSE scores. Besides, hsCRP was negatively correlated with MoCA; while total cholesterol, HDL-C and LDL-C were positively correlated with the MoCA, respectively.

**Conclusion**: Our findings suggest that lower SOD along with cholesterol, HDL-C and LDL-C, and higher hsCRP levels might be important markers to assess the PD severity. A better understanding of SOD and hsCRP may yield insights into the pathogenesis of PD.

## Introduction

Parkinson’s disease (PD) is the second common age-related neurodegenerative disease following Alzheimer’s disease (AD) due to the progressive loss of dopaminergic neurons in the substantia nigra pars compacta (SNpc) and striatum (Zhao et al., 2018; Li et al., 2019; Qian and Huang, 2019). It is clinically characterized by resting tremor, rigidity, bradykinesia, and postural instability (Chomiak et al., 2018; Gao et al., 2018; Ray Chaudhuri et al., 2018; Sun et al., 2018). Increasing evidence points to the importance of neuroinflammation and oxidative stress in the pathophysiology of PD (Adams et al., 2019), but the underlying exact mechanisms remain unclear and need to be elucidated.

Superoxide dismutase (SOD) is one of the most important antioxidant enzymes both inside and outside cell membranes, and it catalyzes the dismutation of superoxide radicals (O_2−_) to hydrogen peroxide (H_2_O_2_), which is converted to water and oxygen by catalase and glutathione peroxidase (Zhu et al., 2019). Some Neurologists have even suggested that SOD is the first-line defense against increased reactive oxygen species (ROS) production in PD patients (de Farias et al., 2016). The onsets of cerebrovascular inflammation are considered as stimuli that trigger or facilitate the development of all kinds of neurological disorders. The excessive inflammatory response can influence blood brain barrier (BBB) integrity, and various inflammation markers have been investigated as potential predictors of PD (Mosley et al., 2006). Among those markers, high-sensitivity C-reactive protein (hsCRP) is one of the most frequently investigated potential biomarkers during the screening and monitoring of inflammatory activity in all kinds of system diseases (Kuo et al., 2005). Previous studies have indicated that high concentrations of hsCRP could increase the paracellular permeability of the BBB, which is associated with increased risk, severity and progression of PD (Nawaz and Mohammad, 2015).

Lipids are molecules that contain fatty acids or a steroid nucleus (e.g., cholesterol) and are the main compositions that constitute cellular membranes, part of membrane rafts and protein anchors, and signaling and transport molecules across BBB (Chen et al., 2018; Shi et al., 2019; Xicoy et al., 2019). A previous study found that dyslipidemia is causally related to BBB impairment and plasma high-density lipoprotein cholesterol (HDL-C) were the lipids most associated with BBB integrity (Bowman et al., 2012; Chen et al., 2018). The association of PD with peripheral blood lipids has been investigated in many studies, but the results are inconsistent. For example, some previous studies found that plasma level of cholesterol was inversely associated with the risk of PD, while others showed opposite tendencies or no significant association (Hu, 2010; Guo et al., 2015; Zhang et al., 2017). Lower plasma levels of HDL-C or no difference in PD patients compared to HCs were also reported. The efficacy of lipid-lowering therapies against neurodegenerative diseases is also controversial (Wei et al., 2013; Xicoy et al., 2019). A previous study has shown that statins, a lipid-lowering drug, may interfere with Aβ metabolism and reduce Aβ-mediated neurodegeneration (Ng and Tan, 2017; Shakour et al., 2019). Whereas, possible adverse effects of intensive lipid-lowering treatment in neurocognitive disorders have been indicated, as cholesterol is a component of the CNS (Mannarino et al., 2018).

Identification of markers in the circulation of PD patients may help us to assess and monitor the disease severity and progression of PD (He et al., 2018). There are evidences suggesting that oxidative stress, lipid metabolism, and inflammatory responses play critical roles in the pathogenesis of PD. Whether oxidative, lipid metabolism and inflammatory mediators could be used as potential biomarkers to detect the severity of PD remains largely unknown. Therefore, we aimed to investigate the value of combined SOD, cholesterol, HDL-C, LDL-C and hsCRP in evaluating and monitoring the severity of PD.

## Materials and Methods

### Subjects and Ethics Statement

This study was approved by the ethics committee of the Zhujiang Hospital of Southern Medical University (2019-KY-071-02) and was conducted according to the principles outlined in the revised Declaration of Helsinki of 1975 and the National Institutes of Health Human Subjects Policies and Guidelines released in 1999. All participants provided written consent to participate in the investigation and allowed investigators to measure their blood sample levels. The PD patients recruited in this present study met the 2015 Movement Disorder Society criteria for the diagnosis of idiopathic PD (Postuma et al., 2015). The exclusion criteria for patients were: The exclusion criteria for patients were: (1) PD patients with other neurological conditions or neuropsychiatric comorbidities, such as cerebrovascular disease or psychiatric disorders (psychosis and severe depression); (2) PD patients with somatic diseases, such as endocrine and malignant diseases, cardiac, hepatic or renal failure, or other life-threatening diseases; (3) PD patients who are taking antilipemic agent, such as statins, fibrates and nicotinic acid; and (4) PD patients who refused to participate in the study. The healthy controls (HCs) subjects were recruited from the Medical Examination Centre of Zhujiang Hospital of Southern Medical University, and those with hypertension, cerebral ischemia, cardiovascular disease, diabetes, psychiatric disorders, hepatic or renal dysfunction were excluded from this study. A total of 204 PD patients (92 males and 112 females) and 204 healthy age- and gender-matched subjects (92 males and 112 females) were recruited in this present study. The control group was created by randomly selecting 1:1 age- and gender-matched subjects.

### Clinical Evaluation and Blood Collection and Measurement

Experienced neurologists performed clinical evaluations, which were conducted in a blinded manner. All PD participants underwent a series of neuropsychological assessments including the unified Parkinson’s disease rating scale (UPDRS; Movement Disorder Society Task Force on Rating Scales for Parkinson’s, 2003) and modified Hoehn and Yahr staging scale (H&Y; Hoehn and Yahr, 1967). UPDRS (I) subscale was applied to measure the mentation, behavior, and mood of PD patients including intellectual impairment, thought disorder, depression and motivation/initiative. UPDRS (II) subscale was applied to measure the daily life functionality of PD patients such as speech, swallowing, handwriting, turning in bed etc. UPDRS (III) subscale was applied to measure the motor dysfunction of PD patients such as hand movements, tremors, rigidity, arising from chair and gait etc. H&Y was used to measure clinical stages and disease progression of PD patients. Mini-Mental State Examination (MMSE; Folstein et al., 1975) and Montreal Cognitive Assessment (MoCA; Smith et al., 2007) were used to measure the cognitive function of PD patients. Venous blood samples from all participants were collected into EDTA tubes by trained nurses in the morning. Blood samples were sent to the clinical pathology department and analyzed immediately, and triple replicates have been performed to assess protein and cholesterol levels. The plasma SOD level was measured with a Cu/Zn SOD ELISA kit (Bio-Techne Corporation, R&D system, Minnesota, MN, USA) according to the manufacturer’s instructions. The hsCRP and lipids levels were measured by latex immunoturbidimetric assay and direct enzymatic methods, respectively.

### Statistical Analysis

All data were analyzed using SPSS 25.0 (IBM Corporation, Armonk, NY, USA). For continuous variables, the results were presented as mean values ± SD. For categorical variables, the results are presented as percentages. When the data were normally distributed, the Student’s *t*-test was performed for two-group comparisons. Correlation coefficients were performed with Spearman’s rank correlation or Pearson correlation analysis to determine the correlation between different clinical parameters. A receiver operating characteristic (ROC) analysis was conducted to assess the performance of clinical markers as discriminative criteria for PD. A value of *P* < 0.05 was considered statistically significant.

## Results

### Demographic and Clinical Characteristics of PD Patients and Healthy Controls

Detailed demographic and clinical characteristics are described in Table 1. A total of 204 PD patients [92 (45.1%) males and 112 (54.9%) females], and 204 healthy control (HC) subjects [92 (45.1%) males and 112 (54.9%) females] were enrolled in this study. The mean ages of the PD patients and HC subjects were 63.94 ± 11.15 and 63.78 ± 11.42 years, respectively. The mean BMI of PD patients and HC subjects were 23.68 ± 3.807 and 24.32 ± 3.290 kg/m^2^, respectively. There were no significant differences in age (*P* = 0.9112) and BMI (*P* = 0.2353) between PD patients and HC subjects. The mean H&Y stage PD patient was 2.385 ± 0.9547. The mean of MoCA and MMSE score for PD patients was 18.40 ± 5.671 and 22.80 ± 4.691, respectively. The mean total UPDRS score was 39.31 ± 18.61, mean UPDRS (I) was 4.348 ± 3.309, mean UPDRS (II) was 14.50 ± 7.180 and mean UPDRS (III) was 21.33 ± 11.05.

**Table 1:** Demographic profiles and clinical characteristics of PD patients and healthy controls.

Clinical parameters		Healthy controls			Patients with PD			PD vs. Control
		mean ± SD	Min	Max	mean ± SD	Min	Max	*P*-value
Gender	Male n (%)	92 (45.10%)	/	/	92 (45.10%)	/	/	/
	Female n (%)	112 (54.90%)	/	/	112 (54.90%)	/	/	/
Age (years)		63.78 ± 11.42	33	91	63.94 ± 11.15	33	91	0.9112
BMI (kg/m^2^)		24.32 ± 3.290	18.36	32.39	23.68 ± 3.807	16.71	32.87	0.2353
Duration (years)		/	/	/	4.780 ± 3.653	0.1	20	/
UPDRS		/	/	/	39.31 ± 18.61	6	108	/
	UPDRS(I)	/	/	/	4.348 ± 3.309	1	21	/
	UPDRS(II)	/	/	/	14.50 ± 7.180	2	48	/
	UPDRS(III)	/	/	/	21.33 ± 11.05	2	54	/
H&Y		/	/	/	2.385 ± 0.9547	1	5	/
MoCA		/	/	/	18.40 ± 5.671	4	28	/
MMSE		/	/	/	22.80 ± 4.691	9	30	/
SOD		147.2 ± 24.34	101.0	208.0	135.7 ± 20.14	68.00	184.0	<0.0001****
hsCRP		1.637 ± 1.597	0.500	11.70	2.766 ± 3.242	0.500	18.00	<0.0001****
UA		352.7 ± 97.55	107.0	660.0	326.9 ± 91.84	139.0	634.0	0.0069**
FN		215.4 ± 36.46	124.7	399.0	211.9 ± 32.32	136.0	327.0	0.3211
RBP		42.25 ± 12.45	13.10	90.00	41.41 ± 11.76	17.00	83.01	0.4942
CysC		0.927 ± 0.207	0.030	1.820	1.036 ± 0.3206	0.630	3.650	<0.0001****
Cr		71.01 ± 17.17	37.60	120.0	74.80 ± 30.85	35.90	312.0	0.1278
Urea		5.325 ± 1.486	2.500	9.500	5.377 ± 1.966	2.140	18.640	0.7634
TG		1.498 ± 1.063	0.290	9.540	1.423 ± 0.8207	0.370	6.460	0.4348
Chol		5.025 ± 0.893	2.490	9.220	4.665 ± 1.024	2.460	8.550	0.0002***
HDL-C		1.435 ± 0.365	0.830	2.550	1.320 ± 0.3494	0.660	2.440	0.0013**
LDL-C		3.051 ± 0.754	1.110	5.160	2.744 ± 0.8474	0.990	5.810	0.0001***

Abbreviations: PD, Parkinson’s disease; BMI, body mass index; UPDRS, Unified Parkinson’s Disease Rating Scale; H&Y, Hoehn, and Yahr staging scale; MoCA, Montreal Cognitive Assessment; MMSE, Mini-Mental State Examination; SOD, superoxide dismutase; hsCRP, high-sensitivity C-reactive protein; UA, urine acid; FN, fibronectin; RBP, retinol-binding protein; CysC, cystatin C; Cr, creatinine; TG, triglyceride; Chol, cholesterol; HDL-C, high-density lipoprotein cholesterol; LDL-C, low-density lipoprotein cholesterol. Data shown are mean ± SD. A comparison of variables between PD and healthy subjects was performed by Student’s t-test. A value of *P* < 0.05 was considered statistically significant. **P < 0.01, ***P < 0.001, ****P < 0.0001.

### Comparisons of Plasma Markers Between PD Patients and Healthy Controls

**Figure 1:**
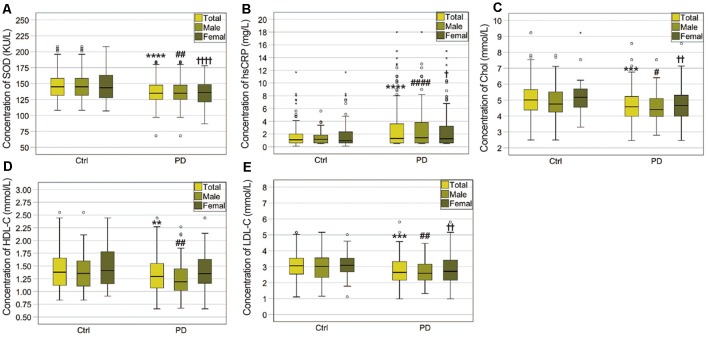
Comparisons of plasma biomarker levels between PD patients and healthy controls (HCs). **(A)** Comparison of superoxide dismutase (SOD) levels between the PD patients and HCs. ****PD (total) vs. Ctrl (total), *P* < 0.0001; ^##^PD (male) vs. Ctrl (male), *P* < 0.01; ^††††^PD (female) vs. Ctrl (female), *P* < 0.0001. **(B)** Comparison of high-sensitivity C-reactive protein (hsCRP) levels between the PD patients and HCs. ****PD (total) vs. Ctrl (total), *P* < 0.0001; ^####^PD (male) vs. Ctrl (male), *P* < 0.0001; ^†^PD (female) vs. Ctrl (female), *P* < 0.05. **(C)** Comparison of cholesterol levels between the PD patients and HCs. ***PD (total) vs. Ctrl (total), *P* < 0.001; ^#^PD (male) vs. Ctrl (male), *P* < 0.05; ^††^PD (female) vs. Ctrl (female), *P* < 0.01. **(D)** Comparison of high-density lipoprotein cholesterol (HDL-C) levels between the PD patients and HCs. **PD (total) vs. Ctrl (total), *P* < 0.01; ^##^PD (male) vs. Ctrl (male), *P* < 0.01. **(E)** Comparison of low-density lipoprotein cholesterol (LDL-C) levels between the PD patients and HCs. ***PD (total) vs. Ctrl (total), *P* < 0.001; ^##^PD (male) vs. Ctrl (male), *P* < 0.01. ^††^PD (female) vs. Ctrl (female), *P* < 0.01.

As shown in Table 1 and Figure 1, significant differences in plasma SOD, hsCRP, cholesterol, HDL-C, and LDL-C levels were observed between PD and HC. Plasma SOD levels of PD patients were remarkably lower than those of HC (135.7 ± 20.14 vs. 147.2 ± 24.34, *P* < 0.0001). PD patients exhibited significantly decreased levels of plasma cholesterol, HDL-C and LDL-C relative to HC (4.665 ± 1.024 vs. 5.025 ± 0.893 for cholesterol, *P* = 0.0002, 1.320 ± 0.3494 vs. 1.435 ± 0.365 for HDL-C, *P* = 0.0013, 2.744 ± 0.8474 vs. 3.051 ± 0.754 for LDL-C, *P* = 0.0001). In addition, plasma hsCRP levels were significantly higher in PD patients than in HC (2.766 ± 3.242 vs. 1.637 ± 1.597, *P* < 0.0001).

When PD patients and HC were divided into specific gender groups, the plasma levels of SOD, cholesterol, and LDL-C in the male and female PD patients were significantly lower than those of HC (Table 2, Figure 1). Plasma HDL-C level in male patients was lower than in female patients (Table 2, Figure 1), while there was no significant difference between female PD patients and female HC. Besides, we found that plasma hsCRP levels in both male and female PD patients were remarkably higher than male and female PD patients (Table 2, Figure 1). Additionally, in PD patients, levels of HDL-C in male patients were lower than in female patients (Table 2), while there were no significant differences in plasma levels of SOD, hsCRP, cholesterol and LDL-C between male and female PD patients. The above results indicated that plasma antioxidant status such as the decreased SOD levels is obviously impaired in PD patients, while plasma levels of inflammatory mediators such as hsCRP are increased.

**Table 2:** Comparisons of SOD, hsCRP, cholesterol, HDL-C, and LDL-C between PD patients and healthy controls according to genders.

Clinical parameters	Gender	Control	PD	PD vs. Control	PD (male vs. female)
		mean ± SD	mean ± SD	*P*-value	*P*-value
SOD	Male	147.9 ± 23.89	137.2 ± 20.80	0.0014**	0.3230
	Female	146.6 ± 24.80	134.4 ± 19.58	<0.0001****
hsCRP	Male	1.402 ± 0.9463	2.963 ± 3.471	<0.0001****	0.4318
	Female	1.831 ± 1.962	2.604 ± 3.047	0.0250*
Chol	Male	4.914 ± 0.9586	4.557 ± 0.9071	0.0104*	0.1729
	Female	5.117 ± 0.8287	4.754 ± 1.107	0.0060**
HDL-C	Male	1.373 ± 0.3308	1.237 ± 0.3171	0.0047**	0.0018**
	Female	1.486 ± 0.3850	1.389 ± 0.3609	0.0529
LDL-C	Male	3.000 ± 0.8401	2.666 ± 0.7620	0.0054**	0.2353
	Female	3.093 ± 0.6754	2.808 ± 0.9101	0.0083**	

Data shown are mean ± SD. A comparison of variables between PD and healthy subjects according to gender was performed by Student’s t-test. A comparison of variables between male and female PD patients was performed by Student’s t-test. A value of *P* < 0.05 was considered statistically significant. *P < 0.05, **P < 0.01, ****P < 0.0001.

### Correlation Analysis Among UPDRS, H&Y, MoCA, MMSE, SOD, hsCRP, Cholesterol, HDL-C and LDL-C Levels in PD Patients

**Figure 2:**
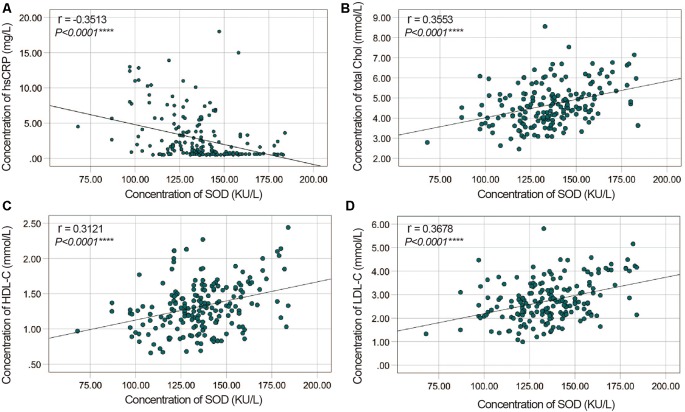
Correlation analysis between plasma SOD and hsCRP/cholesterol/HDL-C/LDL-C in PD Patients. **(A)** A significant negative correlation between SOD and hsCRP in PD Patients. **(B)** A significant positive correlation between SOD and cholesterol in PD Patients. **(C)** A significant positive correlation between SOD and HDL-C in PD Patients. **(D)** A significant positive correlation between SOD and LDL-C in PD Patients. ****P < 0.0001.

**Figure 3:**
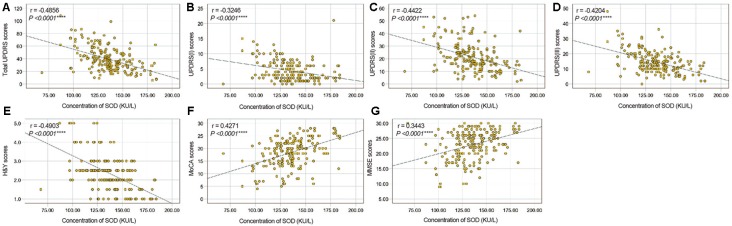
Correlation analysis between plasma SOD and UPDRS/H&Y/MoCA/MMSE scores in PD Patients. **(A)** A significant negative correlation between SOD and total UPDRS scores in PD Patients. **(B)** A significant negative correlation between SOD and UPDRS (I) scores in PD Patients. **(C)** A significant negative correlation between SOD and UPDRS (II) scores in PD Patients. **(D)** A significant negative correlation between SOD and UPDRS (III) scores in PD Patients. **(E)** A significant negative correlation between SOD and H&Y scores in PD Patients. **(F)** A significant positive correlation between SOD and MoCA scores in PD Patients. **(G)** A significant positive correlation between SOD and MMSE scores in PD Patients. ****P < 0.0001.

To evaluate correlations between the disease severity and clinical variables in PD patients, we conducted Pearman’s correlation or Spearman’s rank correlation analysis among mediator variables and various assessments. We found that plasma SOD level was negatively correlated with hsCRP (*γ* = −0.3513, *P* < 0.0001) and positively correlated with cholesterol, HDL-C and LDL-C (*γ* = 0. 0.3553, *P* < 0.0001 for cholesterol, *γ* = 0.3121, *P* < 0.0001 for HDL-C, *γ* = 0.3678, *P* < 0.0001 for LDL-C, Table 3, Figure 2). The plasma SOD level was negatively correlated with UPDRS, UPDRS (I), UPDRS (II), UPDRS (III) and H&Y scores (*γ* = −0.4856, *P* < 0.0001 for total UPDRS; *γ* = −0.3246, *P* < 0.0001 for UPDRS (I); *γ* = −0.4422, *P* < 0.0001 for UPDRS (II); *γ* = −0.4204, *P* < 0.0001 for UPDRS (III); *γ* = −0.4903, *P* < 0.0001 for H&Y; Table 3, Figure 3). While, the plasma SOD level was positively correlated with MoCA (*γ* = 0.4271, *P* < 0.0001) and MMSE (*γ* = 0.3443, *P* < 0.0001). Besides, plasma hsCRP (*γ* = −0.2640, *P* = 0.0001) was negatively correlated with MoCA, while total cholesterol (*γ* = 0.2008, *P* = 0.0040), HDL-C (*γ* = 0.2539, *P* = 0.0002) and LDL-C (*γ* = 0.1665, *P* = 0.0173) were positively correlated with the MoCA, respectively (Table 3). This finding strongly suggests that decreased plasma SOD level is associated with the disease severity in PD patients.

**Table 3:** Correlation analysis of all variables in PD patients.

Variable	SOD		hsCR*P*		Chol		HDL-C		LDL-C]	
	*γ*	*P*	*γ*	*P*	*γ*	*P*	*γ*	*P*	*γ*	*P*
UPDRS	−0.4856	<0.0001****	0.0975	0.1656	−0.1634	0.0195*	−0.1091	0.1204	−0.1300	0.0638
UPDRS(I)	−0.3246	<0.0001****	0.1445	0.0392*	−0.1026	0.1441	−0.0292	0.6787	−0.1057	0.1324
UPDRS(II)	−0.4422	<0.0001****	0.1491	0.0333*	−0.1356	0.0532	−0.1023	0.1452	−0.1380	0.0490*
UPDRS(III)	−0.4204	<0.0001****	−0.0011	0.9870	−0.1318	0.0602	−0.0736	0.2954	−0.1030	0.1427
H&Y	−0.4903	<0.0001****	0.2264	0.0011**	−0.1626	0.0202*	−0.1156	0.0996	−0.1662	0.0175*
MoCA	0.4271	<0.0001****	−0.2640	0.0001***	0.2008	0.0040**	0.2539	0.0002***	0.1665	0.0173*
MMSE	0.3443	<0.0001****	−0.2150	0.0020**	0.1550	0.0268*	0.1217	0.0830	0.1241	0.0769
SOD	/	/	−0.3513	<0.0001****	0.3553	<0.0001****	0.3121	<0.0001****	0.3678	<0.0001****
hsCRP	−0.3513	<0.0001****	/	/	−0.0956	0.1737	−0.1657	0.0179*	−0.0139	0.8435
Chol	0.3553	<0.0001****	−0.0956	0.1737	/	/	0.3771	<0.0001****	0.8761	<0.0001****
HDL-C	0.3121	<0.0001****	−0.1657	0.0179*	0.3771	<0.0001****	/	/	0.2607	0.0002***
LDL-C	0.3678	<0.0001****	−0.0139	0.8435	0.8761	<0.0001****	0.2607	0.0002***	/	/

The Pearson correlation was performed to evaluate the relationship between two continuous variables. The Spearman’s rank correlation was performed to evaluate the relationship between two continuous or ordinal variables. A value of *P* < 0.05 was considered statistically significant. *P < 0.05, **P < 0.01, ***P < 0.001, ****P < 0.0001.

### ROC Curves for SOD, hsCRP, Cholesterol, HDL-C, and LDL-C in the Diagnosis of PD

**Figure 4:**
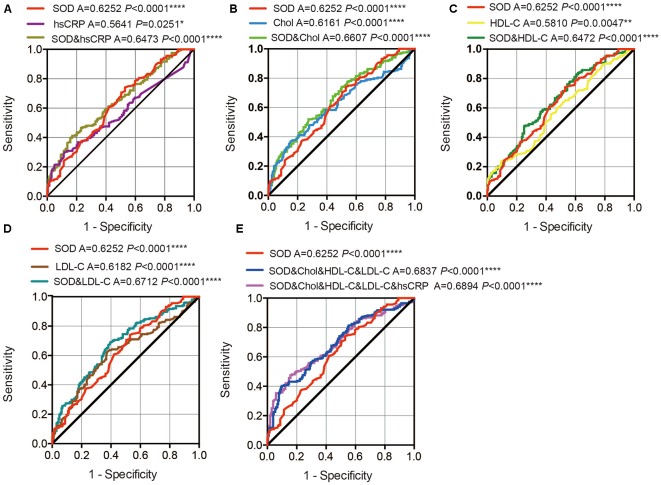
ROC curves to evaluate the utility of plasma levels of SOD, hsCRP, cholesterol, HDL-C and LDL-C for the discrimination of PD patients from healthy controls. **(A)** The area under the curves (AUCs) of the ROC curves for SOD, hsCRP and SOD+hsCRP. **(B)** The AUCs of the ROC curves for SOD, Chol, and SOD+Chol. **(C)** The AUCs of the ROC curves for SOD, HDL-C and SOD+HDL-C. **(D)** The AUCs of the ROC curves for SOD, LDL-C and SOD+LDL-C. **(E)** The AUCs of the ROC curves for SOD, SOD+Chol+HDL-C+LDL-C and SOD+Chol+HDL-C+LDL-C+hsCRP. A, area under the curves. *P < 0.05, **P < 0.01, ****P < 0.0001.

ROC curves were performed to investigate whether plasma SOD, hsCRP, cholesterol, HDL-C, and LDL-C levels could provide potential discrimination between PD patients and HCs. The area under the curve (AUC) value for SOD was 0.6252 (95% CI: 0.5715–0.6790, *P* < 0.0001), the cut-off was 145.7, with a sensitivity of 47.1% and a specificity of 74% (Figure 4). AUC for hsCRP was 0.5641 (95% CI: 0.5081–0.6202, *P* = 0.0251), and cut-off was 3.05, with a sensitivity of 29.9% and a specificity of 88.2% (Figure 4A). The AUC for cholesterol was 0.6161 (95% CI: 0.5613–0.6708, *P* < 0.0001), the cut-off was 4.21, with a sensitivity of 84.8% and a specificity of 36.8% (Figure 4B). The AUC for HDL-C was 0.5810 (95% CI: 0.5258–0.6362, *P* = 0.0047), and the cut-off was 1.05, with a sensitivity of 90.2% and a specificity of 24% (Figure 4C). The AUC for LDL-C was 0.6182 (95% CI: 0.5635–0.6729, *P* < 0.0001), and the cut-off was 2.86, with a sensitivity of 62.7% and a specificity of 62.3% (Figure 4D). These data indicate that hsCRP, SOD, cholesterol, HDL-C and LDL-C alone have a diagnostic value in distinguishing PD patients from HC.

In addition, we also conducted a ROC analysis for the combination of SOD and hsCRP/cholesterol/HDL-C/LDL-C levels in the discrimination between PD patients and HCs. The AUC for SOD and hsCRP was 0.6473 (95% CI: 0.5945–0.7000, *P* < 0.0001 0.06107), with a sensitivity of 41.2% and a specificity of 82.8%, and the cut-off was 0.57, depending on the predicted risk algorithm (Figure 4A). The AUC for SOD and cholesterol was 0.6607 (95% CI: 0.6084–0.7130, *P* < 0.0001), with a sensitivity of 52% and a specificity of 72.5%, and cut-off was 0.54 (Figure 4B). The AUC for SOD and HDL-C was 0.6472 (95% CI: 0.5944–0.7000, *P* < 0.0001), with a sensitivity of 47.5% and a specificity of 75%, and the cut-off was 0.57 (Figure 4C). The AUC for SOD and LDL-C was 0.6712 (95% CI: 0.6191–0.7233, *P* < 0.0001), with a sensitivity of 69.1% and a specificity of 60.3%, and the cut-off was 0.49 (Figure 4D). When we combined the SOD, cholesterol, HDL-C and LDL-C values, the AUC was 0.6837 (95% CI:0.6323–0.7351, *P* < 0.0001), with a sensitivity of 40.2% and a specificity of 90.2% at the cut-off of 0.60 (Figure 4E). When we combined the SOD, cholesterol, HDL-C, LDL-C and hsCRP values, the AUC was 0.6894 (95% CI: 0.6382–0.7405, *P* < 0.0001), with a sensitivity of 47.5% and a specificity of 84.3% at the cut-off of 0.58 (Figure 4E). These data indicate that the combination SOD with cholesterol, HDL-C, LDL-C and hsCRP levels was more robust than SOD, cholesterol, HDL-C and LDL-C alone in distinguishing PD patients from HC.

## Discussion

In this present study, we explored the variations of plasma levels of SOD, hsCRP, cholesterol, HDL-C and LDL-C in PD patients and noted several interesting results. First, we found a pronounced decrease in the plasma levels of SOD, cholesterol, HDL-C, and LDL-C and an increase in the plasma level of hsCRP in PD patients when compared to HC. Second, the plasma level of SOD was negatively correlated with the plasma level of hsCRP but positively correlated with the plasma levels of cholesterol, HDL-C and LDL-C in PD patients. Third, there were significant correlations between the decreased plasma SOD level and the disease severity of PD. The interesting finding of an inversed correlation between plasma levels of SOD and hsCRP may shed light on the underlying neuro-pathogenesis of PD.

More and more evidence indicates that BBB dysfunction contributes to the pathophysiological process of PD (Iadecola, 2017). A post-mortem study of advanced PD cases showed vessel degeneration in multiple brain regions, especially in the substantia nigra, middle frontal cortex and brain stem nuclei (Guan et al., 2013; Zheng et al., 2018; Calvo-Rodriguez et al., 2019; Wei et al., 2019). Other studies also demonstrated that BBB permeability was increased in the late stages of PD (Kortekaas et al., 2005; Pisani et al., 2012). Since the BBB was impaired, various neurotoxic molecules and red blood cells (RBCs) can leak from peripheral circulation into brain parenchyma. RBCs invasion causes accumulation of hemoglobin-derived neurotoxic products including iron, which generates neurotoxic ROS (Zlokovic, 2011; Cai et al., 2017). Dopaminergic cells are equipped with abundant amounts of mitochondria, and therefore more vulnerable to ROS attacks (Calvo-Rodriguez et al., 2019). SOD, as one of the most important antioxidant enzymes, is supposed to be the first-line defense against increased ROS production in PD (Peng et al., 2005; Botella et al., 2008; de Farias et al., 2016). The decrease in SOD reduces the scavenging of O_2−_ and the balance was shifted towards oxidative stress in PD patients, which eventually lead to the development of PD. In this study, we found that the plasma level of SOD in PD patients was remarkably lower than HC, indicating that lower plasma levels of SOD may contribute to the neuro-pathogenesis of PD, and may be correlated to the disease severity. Our finding is in agreement with the results of previous studies. Gruden et al. (2012) demonstrated that the plasma activity of SOD in PD patients was diminished. Yuan et al. (2016) found that plasma activity of SOD in *de novo* Chinese PD patients were lower than in HCs. Furthermore, Li et al., 2017 generated MPTP-treated PD monkey model and found that serum SOD showed continuously decrease. However, there are also some contradictory results in PD patients showing increased SOD in the peripheral blood when compared with HC (Serra et al., 2001; Sharma et al., 2008; de Farias et al., 2016). Whereas in some other studies, although a slight decrease in PD was indicated, they did not find a statistical difference in plasma SOD between PD patients and HC (Watfa et al., 2011). The significant heterogeneity among those studies might be due to the different ethnic groups, disease severity, disease duration, medication status, limited number of subjects. Therefore, we specifically examined the relationship between plasma SOD level and PD severity. In this study, we found that the plasma SOD concentration was significantly negatively correlated with the scores of UPDRS, UPDRS (I), UPDRS (II), UPDRS (III), and H&Y. UPDRS (I) subscale was applied to measure the mentation, behavior and mood including intellectual impairment, thought disorder, depression and motivation/initiative. UPDRS (II) subscale was applied to measure the daily life functions such as speech, swallowing, handwriting, turning in bed etc. UPDRS (III) subscale was applied to measure the motor dysfunction such as hand movements, tremors, rigidity, arising from chair and gait. Previous study has also demonstrated that SOD change was closely related to the appearance of clinical symptoms in MPTP-treated monkeys such as akinesia, rigidity and tremor (Li et al., 2017). Furthermore, we noted the significantly positive correlation between plasma SOD level and MoCA/MMSE scores in PD patients, indicating that decreased plasma SOD level in PD might, at least partially, correlate to the cognitive impairment of PD patients. Previous studies have also demonstrated that higher SOD level is associated with a decreased risk of dementia (Casado et al., 2008). Based on our findings, the decreased SOD might be an important marker to assess the disease severity of PD patients.

Lipids are molecules that contain fatty acids or a steroid nucleus (e.g., cholesterol). They are the main compositions that constitute cellular membranes, part of membrane rafts and protein anchors, and signaling and transport molecules across BBB (Shi et al., 2019). Several lines of evidence already indicated that abnormalities in lipid metabolism are involved in the pathophysiological process of PD (Chung et al., 2019; Xicoy et al., 2019). A previous study demonstrated that dyslipidemia is causally related to BBB impairment and plasma HDL cholesterol were the lipids most associated with BBB integrity (Bowman et al., 2012; Deng et al., 2018; Eiden et al., 2019; Miao et al., 2019). α-synuclein is a small protein abundantly expressed in the brain. It is well-established that the folding and aggregation of α-synuclein is a pathological hallmark of Parkinson’s disease. Previous study indicated that the disruption of lipids homeostasis may lead to the accumulation of α-synuclein *via* a direct lipid-protein interaction (Galvagnion, 2017). In this present study, we found that plasma levels of cholesterol, HDL-C and LDL-C were significantly decreased in PD patients compared to HC. Our findings are in agreement with previous studies, showing lower levels of plasma total cholesterol, LDL-C, HDL-C in PD patients compared with HCs (Guo et al., 2015; Zhang et al., 2017). Fang et al. (2019) found that higher levels of total cholesterol, LDL-C, and triglycerides were associated with a lower future risk of PD. Furthermore, previous studies indicated that a higher plasma level of cholesterol has been associated with reduced PD risk and slower clinical progression of PD (Huang et al., 2011, 2015; Rozani et al., 2018). cholesterol intake has been suggested to be negatively correlated with PD risk (Powers et al., 2009). Lower plasma level of HDL has also been found to be associated with earlier PD onset and higher PD risk (Qiang et al., 2013; Lu et al., 2014; Swanson et al., 2015a,b). Lower level of LDL-C was reportedly associated with higher PD risk, while higher level of LDL-C might be protective for PD and associated with preserved executive and fine motor functions in PD (Huang et al., 2008; Du et al., 2012; Rozani et al., 2018). These results suggested that the disruption of lipids homeostasis may be involved in the pathogenesis of PD.

Interestingly, we noticed a significant and positive correlation between SOD and cholesterol, HDL-C and LDL-C in PD patients. It has been shown that increased cholesterol, either *in vivo* in cholesterol-fed wt mice or *in vitro* in genetically induced cholesterol accumulation due to NPC1 loss-of-function, could potentiate SOD activity, suggesting that SOD might be a prime target and the first defense mechanism of oxidative stress response upon increased cholesterol (Dominko et al., 2018). Multiple lines of evidence already indicate that a high level of LDL-C could cause the free radical formation and excessive oxidative stress, which initially leads to a progressive increase in endogenous enzymatic scavengers like SOD (Gupta et al., 2009). Therefore, we propose that both SOD, cholesterol, HDL-C and LDL-C would directly participate in the pathophysiological mechanism of PD.

Evidence is now overwhelming that inflammatory responses manifested by glial reactions, increased expression of inflammatory cytokines, and other toxic mediators are important contributors to the progressive loss of nigral dopaminergic (DA) neurons in PD patients. Previous studies found that the aging process can exaggerate the inflammatory responses and aggravate the DA neurons loss in the brain (Zhao et al., 2018). Additionally, the existence of ongoing inflammatory processes affects the modulation of neurogenesis, for example, TNFα affects the differentiation and proliferation of neural stem cells by up-regulation of Ascl2 (Liu et al., 2019). Previous studies demonstrated that systemic inflammation may exacerbate the symptoms of chronic neurodegenerative diseases, indicating that systemic inflammation may promote neurodegeneration (Cunningham et al., 2009; Perry, 2010). The level of hsCRP in the peripheral blood is a well-studied non-specific marker of low-grade systemic inflammation. Previous studies have indicated that high concentration of hsCRP may disrupt BBB (Schmidt et al., 2002) and increase the paracellular permeability by binding to the Fcγ receptor, which results in the entrance of hsCRP and other peripheral inflammatory proteins into the central nervous system and eventually leads to reactive gliosis and development of PD (Wilms et al., 2007; Chen et al., 2008). In this case, the level of hsCRP in the peripheral blood may reflect the neuro-inflammatory status in the central nervous system. In the present study, we found a pronounced increase in the plasma level of hsCRP in PD patients compared to HC, which is in line with previous reports (Baran et al., 2019). Besides, in this present study, we found that plasma hsCRP level was negatively correlated with MoCA in PD, suggesting that elevated hsCRP level was associated with poor cognitive performance. Our finding is in accord with previous studies that higher level of hsCRP was associated with cognitive decline in the general population and AD (Kuo et al., 2005; Komulainen et al., 2007; Marioni et al., 2009; Xu et al., 2009). Several underlying mechanisms might explain the involvement of hsCRP in the process that contributes to cognitive impairment in PD patients. Accumulating evidence suggests that inflammation is associated with cardio-cerebrovascular diseases (Kuo et al., 2005; Wichmann et al., 2014). The hsCRP, rather than just simply acting as a systemic inflammatory marker, is also a proatherogenic role and involved in atherogenesis through the mediation of LDL uptake by macrophages, facilitation of foam-cell formation and low nitric-oxide production, stimulation of monotype recruitment and vascular smooth muscle proliferation, thus leading to cerebral microangiopathy and macroangiopathy. All of these finally would result in the disruption of the frontal-subcortical circuit and cognitive impairment (Kuo et al., 2005). These results strongly imply that the plasma hsCRP level could be used to assess cognitive impairment in PD patients.

Interestingly, in this present study, our findings indicated the decreased SOD in PD, and we proposed that this decrease might contribute to the cognitive impairment in PD patients; while the increased hsCRP might aggravate cognitive impairment in PD patients. This contra-directional effect of SOD and hsCRP in cognitive function indicating they might perform the opposite function in PD patients. The hsCRP not only performs an important role in the inflammatory mechanisms but also induces oxidative stress. Kobayashi et al., 2003 found that hsCRP could induce the generation of ROS in cultured coronary artery smooth muscle cells by enhancing p22phox protein expression. Venugopal et al. (2003) showed that hsCRP could stimulate O_2−_ release and resulted in decreased prostacyclin release in human aortic endothelial cells. On the other hand, O_2−_ has involved not just oxidative stress but also pro-inflammatory roles, which may contribute to the recruitment of neutrophils to sites of inflammation, the formation of chemotactic factors, cytokine release, neurotransmitter and hormone inactivation, adhesion molecules expression, lipid peroxidation and oxidation and DNA damage. Previous studies also indicated that O_2−_ could activate nuclear factor-κB, which finally induced hsCRP expression (Zhao et al., 2019). SOD is the major antioxidant enzyme in the human antioxidant system to protect against oxidative stress, which catalyzes the dismutation of highly reactive O_2−_ to less reactive H_2_O_2_ and oxygen. Therefore, SOD can be considered to be anti-inflammatory and anti-oxidative stress. Indeed, SOD mimetic has been successfully used to prevent the extent of inflammation (De Lazzari et al., 2018; Ren et al., 2018). As mentioned above, SOD is the prime target and the first defense mechanism of oxidative stress response upon increased cholesterol /LDL-C (Gupta et al., 2009; Dominko et al., 2018), which may prevent the inflammatory response caused by cholesterol /LDL-C to some extent. Therefore, there seems to be a strong and complicated interaction between hsCRP/cholesterol/LDL-C pro-inflammatory function and SOD anti-inflammatory function, which may influence the cognitive dysfunctions in PD patients.

There are several limitations to this study: (1) a relatively small number of participants (204 PD patients and 204 healthy subjects) were recruited. In the future, a large number of participants would be recruited; (2) this study is a cross-sectional study. Therefore, longitudinal cohort studies with a larger population need to be performed in the future; and (3) absence of the assessment of other inflammatory markers like cytokines. Furthermore, the indexes of oxidative stress and inflammation status should be further investigated to comprehensively understand the roles of oxidative stress and inflammation in PD.

In summary, plasma antioxidant status such as the SOD is obviously impaired in PD patients, while plasma inflammation such as hsCRP is increased. This finding supports the idea that oxidative stress and inflammation in the peripheral blood may contribute to the pathogenesis of PD. Decreased SOD along with cholesterol, HDL-C and LDL-C and increased hsCRP might be important markers to assess the disease severity of PD patients. Further prospective studies are needed to validate these findings that will help us better understand PD pathogenesis and potential neuroprotection effects of SOD.

## Data Availability Statement

All datasets generated for this study are included in the article.

## Ethics Statement

The studies involving human participants were reviewed and approved by the ethics committee of the Zhujiang Hospital of Southern Medical University. The patients/participants provided their written informed consent to participate in this study.

## Author Contributions

WY, SZ, and QW conceived and designed the study. WY, ZC, RQ, GW, BD, ZH, FX, XW, QY, and TW performed the study. ML, KM, BT, VM, and FZ revised the article for intellectual content. WY and ZC performed data statistics and analysis. WY and QW wrote the article. All authors read and approved the final manuscript.

## Conflict of Interest

The authors declare that the research was conducted in the absence of any commercial or financial relationships that could be construed as a potential conflict of interest.

## Abbreviations

AD: Alzheimer’s disease
BMI: body mass index
Chol: cholesterol
HDL-C: high-density lipoprotein cholesterol
H&Y: Hoehn and Yahr scale
H_2_O_2_: hydrogen peroxide
hsCRP: high-sensitivity C-reactive protein
LDL-C: low-density lipoprotein cholesterol
MMSE: Mini-Mental State Examination
MoCA: Montreal Cognitive Assessment
O_2−_: superoxide radicals
PD: Parkinson’s disease
ROC: receiver operating characteristic
ROS: reactive oxygen species
SNpc: substantia nigra pars compacta
SOD: superoxide dismutase
UPDRS: Unified PD rating scale.
